# *Exiguobacterium* sp. is endowed with antibiotic properties against Gram positive and negative bacteria

**DOI:** 10.1186/s13104-021-05644-2

**Published:** 2021-06-08

**Authors:** Nicole T. Cavanaugh, Anutthaman Parthasarathy, Narayan H. Wong, KayLee K. Steiner, Jonathan Chu, Joseph Adjei, André O. Hudson

**Affiliations:** 1 Thomas H. Gosnell School of Life Sciences, Rochester Institute of Technology, 85 Lomb Memorial Drive, Rochester, NY 14623 USA; 2 National Technical Institute for the Deaf, Rochester Institute of Technology, 52 Lomb Memorial Drive, Rochester, NY 14623 USA

**Keywords:** Antibiotic resistance, *Exiguobacterium*, Whole-genome sequencing, Secondary metabolite, Antibiotics

## Abstract

**Objective:**

In order to isolate and identify bacteria that produce potentially novel bactericidal/bacteriostatic compounds, two ponds on the campus of the Rochester Institute of Technology (RIT) were targeted as part of a bioprospecting effort.

**Results:**

One of the unique isolates, RIT 452 was identified as *Exiguobacterium* sp. and subjected to whole-genome sequencing. The genome was assembled and in silico analysis was performed to predict the secondary metabolite gene clusters, which suggested the potential of *Exiguobacterium* RIT452 for producing antibiotic compounds. Extracts of spent growth media of RIT452 were active in disc diffusion assays performed against four reference strains, two Gram-negative (*E. coli* ATCC 25922 and *P. aeruginosa* ATCC 27853) and two Gram-positive (*B. subtilis* BGSC 168 and *S. aureus* ATCC 25923). Differential extraction and liquid chromatography was used to fractionate the extracts. Efforts to identify and elucidate the structure of the active compound(s) are still ongoing.

**Supplementary Information:**

The online version contains supplementary material available at 10.1186/s13104-021-05644-2.

## Introduction

Antibiotic resistance was first discovered in the 1940s, when it was observed that bacteria were become increasingly resistant to measures taken against them [1]. However, this development did not become a global issue until antibiotic abuse in medicine and animal husbandry became more widespread [2–4]. Antibiotic resistance dramatically increased in the 1980s and 1990s due to a discovery void. The progressive increase of antibiotic resistance and the decline in research and development of antibiotics combined to produce the current crisis [5]. Since there have been few developments in the creation of new antibiotic classes for many years, bacteria are becoming increasingly resistant to those that are currently in use [1, 6]. The targets of antibiotics are diverse; protein synthesis, DNA replication, RNA synthesis and the cell membrane are common targets [4]. Antibiotics with novel targets need to be developed for replacing the existing drugs.

The β-lactam core structure is part of a number of antibiotic classes, but β-lactam resistance is widespread and is conferred by over 300 types of β-lactamases, of which 200 are able to hydrolyze extended spectrum cephalosporin antibiotics [7]. Therefore, increasing the structural diversity of antimicrobial compounds becomes important. Estimates of all possible small molecules are pegged at 10^63^ distinct molecules, of which many may not be accessible by synthesis [8]. Only a small fraction of these molecules are biologically relevant [9]. However, natural compounds offer better chances of finding bioactive molecules with entirely new chemical scaffolds than combinatorial chemistry libraries, since they have evolved to be bioactive, and can often enter cells via transmembrane transporters rather than by passive diffusion [10]. Statistical analysis reveals the continuing trend of chemical novelty in natural products as de-replication tools are increasingly being implemented [11]. In this context, shifting to less known bacterial phyla, rather than over-represented phyla such as the Actinobacteria might increase diversity of the antibiotics identified by prospecting [12]. The goal of our study is to identify novel bacteria that yield potentially novel compounds, which may become starting points to develop more specific and potent antibiotics.

RIT452 was identified during the screening of bacterial isolates from a campus pond, with potential bacteriostatic/bactericidal properties, and its genome was sequenced. The genome was subjected to secondary metabolite analysis via the antibiotics and secondary metabolite analysis shell 5.0 (antiSMASH) [13] and the antibiotic resistance target seeker (ARTS) platforms [14]. Organic compounds extracted from the spent growth media of RIT452 were shown to inhibit the growth of Gram negative and Gram positive reference strains.

The data presented here shows that *Exiguobacterium* sp. RIT452 produces a broad spectrum antibiotic, which after fractionating by differential extraction and chromatography, was not structurally characterized. The facultative anaerobic Gram-positive genus *Exiguobacterium* consists of motile, non-spore forming species [15], widely distributed in the environment, including extremophiles growing in high altitude salt plains [16], hot springs [17, 18], oceans, Antarctic dry valleys and permafrost [18], while others tolerate gamma radiation [19], organic solvents [20], chromium and mercury [21–23], arsenic [16, 24–26], pesticides [27], alkaline wastewater [28], and heat [29–32]. All of this is accomplished without the ability to form spores, suggesting other physiological changes help them cope with these stresses.

## Main text

### Methods

#### Bacterial growth and characterization

Tryptic soy broth (TSB) cultures were used for DNA extraction, while LB (Lysogeny broth) was used for the antibiotic production cultures. R2A (Reasoner’s 2A) minimal media were used for starvation experiments according to Yang et al. [33]. Tryptic soy agar (TSA)-grown cells were analyzed after weeks 1 and 5 by electron microscopy to examine the morphological changes. RIT452 was identified as an *Exiguobacterium* sp. based on the 16S rRNA gene sequence. The V3/V4 region was sequenced by Sanger nucleotide sequencing (GeneWiz LLC, South Plainfield, NJ) and was analyzed by the basic local alignment search tool (BLAST) [18].

#### PCR amplification and nucleotide sequencing of the 16S V3/V4 regions

Bacteria from ponds at the Rochester Institute of Technology (RIT) were isolated on TSA or R2A media. Each was subjected to PCR using primers, 5′-CCTACGGGNGGCWCGAG-3′ (forward) and 5′-GACTACHVGGGTATCTAATCC-3′ (reverse) designed to amplify the V3/V4 rRNA regions. The following thermal cycler protocol was used: 1 cycle at 95 °C for 2 min, 30 cycles each at 95 °C for 30 s, 52 °C for 30 s and 72 °C for 3 min, 1 cycle at 72 °C for 5 min, and finally infinite hold at 4 °C. The PCR products were separated by gel electrophoresis, followed by Sanger nucleotide sequencing of the amplified sequences (GeneWiz LLC, South Plainfield, NJ) prepared with the V3/V4 forward primer.

#### Genomic DNA isolation, library preparation, genome sequencing and alignment

RIT452 grown in 3 mL of TSB for 24 h at 30 °C. The DNA isolation and subsequent steps were performed as in Steiner et al. [34].

#### Predicting secondary metabolite production

The aligned genome sequence of *Exiguobacterium* sp. RIT 452 was analyzed using the antibiotics and secondary metabolite analysis shell (antiSMASH4.0) webserver [13]. The aligned genome was also analyzed by the antibiotic resistance target seeker (ARTS version 2) webserver [14]. ARTS predicts resistance mechanisms and BGCs from genome sequences [14].

#### Extraction of organic compounds from culture media

RIT 452 was cultured in 100 mL LB medium at 30 °C shaken at 130 rpm for 24 h. This was inoculated in 900 mL of LB medium and grown for an additional 48 h at 30 °C shaken at 130 rpm. The extraction of organics from the spent media and storage of concentrated extracts were performed as published earlier [34].

#### Compound fractionation and liquid chromatography (LC)

A five-step extraction and the subsequent separation of the most active extract by liquid chromatography were conducted using known methods [34].

#### Broth dilution assay vs. clinical pathogens

Three clinical pathogens were used: MRSA USA300-FPR3757 (*mecA*) [35], *E. coli* MCR1_NJ [*mcr-1, bla*_NDM-5_*, strA, strB, aac*(6*′*)*-Ib-cr, bla*_OXA-1_*, arr-3, sul1, sul2, tet*(A)] [36], and *P. aeruginosa* AR-0230 (*aac*(3)*-Id*, *aadA2*, dfrB5, OXA-4, OXA-50, tet(G), VIM-2) [37]. Briefly, experiments were conducted in cation adjusted Mueller Hinton broth and a starting bacterial inoculum of  ~ 10^6^ cfu/mL for each isolate. The minimum inhibitory concentration (MIC) for the crude extract against all three clinical isolates, determined using broth micro-dilution according to the Clinical and Laboratory Standards Institute (CLSI) Guidelines, was 6.25 ×  (equivalent to metabolites extracted from 6.25 mL of spent media) [38].

#### Disc diffusion assays

Disc diffusion tests of the organics extracted with different solvents was performed according to Steiner et al. [34].

#### Disc diffusion assays of LC fractions

The activity was tested against *E. coli* ATCC 25922, *P. aeruginosa* ATCC 27853, *B. subtilis* BGSC 168 and *S. aureus* ATCC 25923. The activity was better against the Gram positive strains (Additional file 1: Figure S1). Further enrichment of the active compounds was performed with *S. aureus* ATCC 25923 as the test strain. *S. aureus* ATCC 25923 was grown overnight in 5 mL LB medium at 30 °C in a 130 rpm shaker incubator. The cells were pelleted and each culture was re-suspended in 2 mL of sterile PBS. 180 μL of the re-suspension was mixed into tubes containing 40 ml of warm LB agar and poured into square petri dishes. The petri plates were cooled for 1 h in a sterile hood. 6 mm sterile blank paper disks (BD Biosciences, USA) were aseptically placed onto each agar plate. Methanol (20 μL), tetracycline stock (10 mg/mL, 22.5 μM, 20 μL), and each fraction (60 μL of each fraction re-suspended in 100 μL methanol) were pipetted onto the disks. The plates were dried aseptically for 1 h and incubated for 16 h at 30 °C. The diameter of each zone of inhibition (ZOI) was measured in mm.

#### Flow injection analysis (FIA) and liquid chromatography-mass spectrometry (LCMS)

Low resolution mass spectrometry data was obtained on an Agilent LC/MSD VL system by electrospray ionization (ESI) flow injection analysis in the (positive or negative) mode at the Boston University Chemical Instrumentation Center. A reverse-phase C18 Zorbax Eclipse 2.1 × 50 mm column (Agilent) was used, and the mobile phases were water and acetonitrile with 0.1% formic acid. Separation was achieved by a flow rate of 0.15 mL/min and a mobile phase gradient from 5 to 95% acetonitrile in 10 min. The MS settings were: voltage  =  3000 V, fragmentor  =  70 and chamber temperature  =  350 °C.

#### Scanning electron microscopy (SEM)

The microbiological sample preparation followed an open source protocol [39]. Samples were covered with gold–palladium for 2 minutes with an SPI sputter coater to mitigate charging in the electron beam. The SEM was performed at 5 kV using a Mira3Tescan field emission SEM at the Rochester Institute of Technology (RIT) Nano-Imaging Lab.

## Results

### Strain characterization and phylogeny

The genome was sequenced using an Illumina MiSeq. After assembly, the genome sequence has been deposited in GenBank under accession number QXJB00000000 (BioProject number PRJNA489292; BioSample number SAMN09954399).

### Electron microscopy analysis

**Fig. 1:**
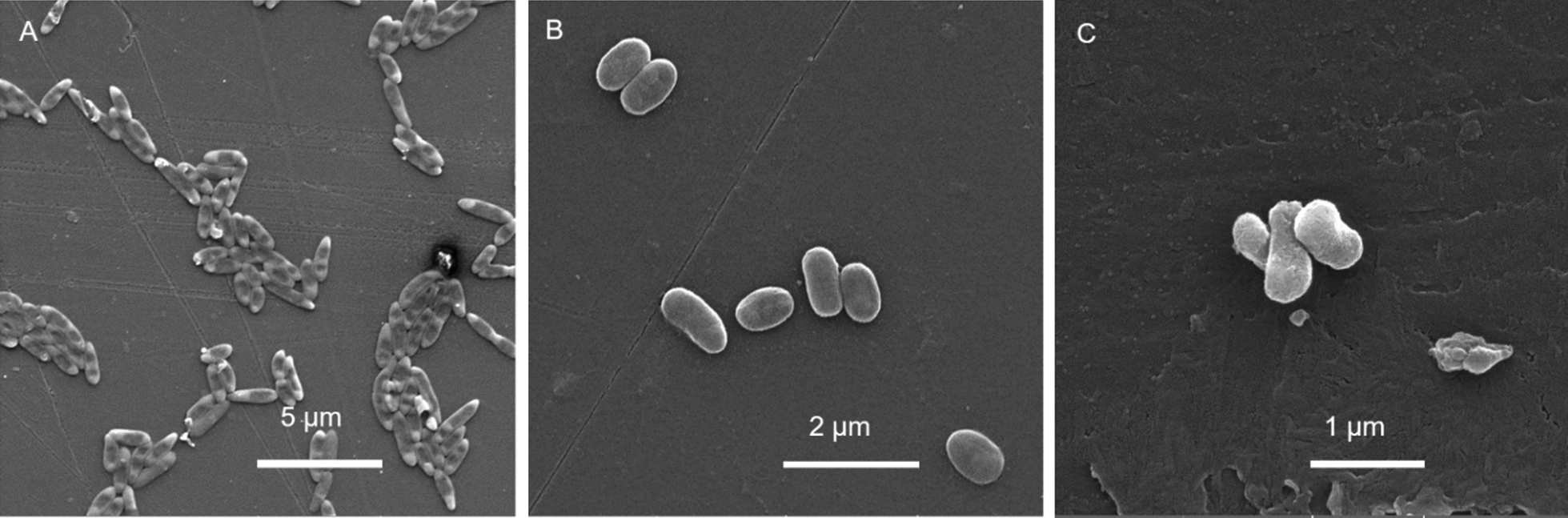
SEM analysis. *Exiguobacterium* RIT452 under different conditions. **A** Cell elongation observed due to starvation on R2A (minimal medium); **B** cells grown on TSA for 1 week and **C** cells grown on TSA for 5 weeks. The magnifications are 9830 ×, 26,500 ×, and 44,900 ×, respectively

RIT452 grown on solid media under different conditions and Fig. 1 shows the scanning electron microscopy (SEM) images recorded for each. In minimal media [33], the cells grow longer, closer to 2 µm, and form larger aggregates (Fig. 1A). Healthy cells are around 1 µm in length and around 0.5 µm wide (Fig. 1B); However when healthy cells are grown for prolonged periods in rich broth media, the cell surface alters and appears rougher (Fig. 1C).

### antiSMASH and ARTS results

The antiSMASH 5.0 web tool analyzes genome sequences and predicts the secondary metabolites produced by an organism [13]. antiSMASH predicted that RIT452 contained four biosynthetic gene clusters (BGCs), two terpene clusters, a siderophore cluster, and a putative antibiotic cluster (Table 1). The “putative antibiotic” BGC identified at locus 897206–908755 shares sequence similarity with a Lugdunin locus, an antibiotic first isolated from *S. lugdunensis*, a commensal of the human nasal cavity [40]. This novel thiazolidine-containing cyclic peptide antimicrobial was shown to be effective against *S. aureus.* Using the genome sequence of RIT 452, ARTS predicted three BGCs, out of which two show “proximity hits” (Table 1). The “proximity hits” readout in the ARTS program shows scaffold-specific self-resistance [14], which is considered a good way to mine for novel antibiotic BGCs [41].

**Table 1:** Secondary metabolites predicted by antiSMASH 5.0 genome mining

Cluster no.	Predicted biosynthetic metabolite	Coordinates within the genome	% Similarity to known cluster	ARTS BGC proximity hits
1	Terpene	69,899–90,726	33 (with carotenoid biosynthetic gene cluster)	–
4	Siderophore	5,05,835–5,19,165	–	Yes
9	Putative antibiotic	8,97,206–9,08,755	26 (with lugdunin biosynthetic gene cluster)	
20	Terpene	1,60,337–1,81,161	–	Yes

The compounds shown in this table show four of the 24 compounds antiSMASH predicted, which could potentially exhibit antibiotic characteristics. If core and resistance genes have intersecting locations on the same scaffold as predicted by ARTS, they are marked as BGC proximity hits

### Broth dilution assays

The crude extract from 1 Liter of spent RIT 452 media was able to inhibit the pathogens MRSA, *E. coli* ncr1_NJ, and *P. aeruginosa* AR-230 at moderate concentrations and the apparent minimum inhibitory concentrations (MIC) in each case is shown in the Additional file 1: Table S1.

### Disc diffusion assays

Crude ethyl acetate extract from spent LB media was spotted on sterile discs and plated on LB agar seeded with different species of bacteria which are not clinical pathogens, whereby the extract inhibited the growth of all strains tested (Additional file 1: Figure S1). The zones of inhibition (ZOIs) show a graded increase with increased volume of extract. The dose response curve created using the ZOIs shows that the extract inhibited the growth of *S. aureus, E. coli,* and *P. aeruginosa* along roughly the same trend while *B. subtilis* had a steeper increase in dosage response (Additional file 1: Figure S1). Among the crude extracts obtained by processing with hexanes, toluene, ether, dichloromethane and ethyl acetate, only one fraction (diethyl ether) showed a ZOI.

### Enrichment of active metabolites using liquid chromatography

**Fig. 2:**
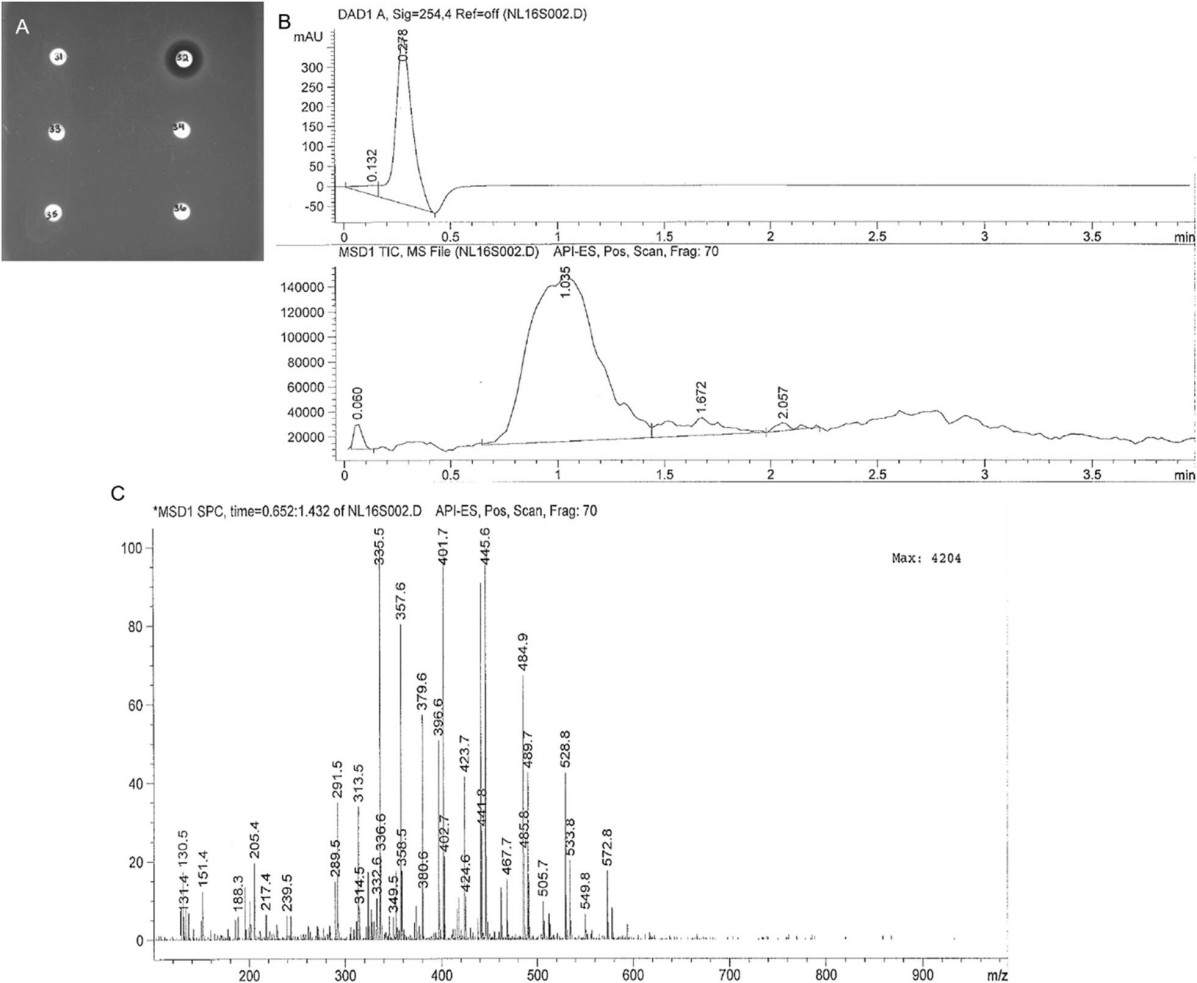
Disc assay using LC fractions 31–36 from diethyl ether extraction and LCMS data. **A** The LC protocol was run to collect fractions of the RIT452 diethyl ether extract. Each was condensed, fully dried, and re-suspended in 100 µl of methanol. 60 µl of each fraction was added to a sterile diffusion disc and plated on LB agar seeded with *S. aureus*. The zone of inhibition (ZOI) of fraction 32 is 15 mm after 16 h of growth. **B** The LC channel in the LCMS shows only one chromatographic peak, while the MS channel shows at least four ionizable species in the positive scan mode. **C** The MS pattern corresponding to the beginning of the largest MS peak (0.652 min) in **B** shows the peaks 335.5, 401.7 and 445.6 in the positive scan mode between 100 and 1000 m/z

The diethyl ether extract when subjected to liquid chromatography using a C18 column (see methods) with *S. aureus* as the test strain. The extract when fractionated showed activity only in fraction 32 (ZOI  =  15 mm), corresponding to a single peak in the LC channel of the LCMS (Fig. 2A), but at least four ionizable species in the positive scan mode (Fig. 2B). The largest peak corresponding to 0.652 min shows the masses 335.5, 401.7 and 445.6 in the positive scan mode. The active compound(s) were not further characterized.

## Discussion

*Exiguobacterium* strains have been previously reported to nearly triple their cell length after exposure to organic solvents [20], whereas RIT452 also undergoes cell elongation upon starvation. This suggests that cell elongation might be a generic stress response in the *Exigobacterium* genus. However, it is not known if this response also occurs as a result of interaction with other bacteria.

An Indian rhizosphere *Exiguobacterium* strain produces a broad spectrum with an unusual dihydroergotamine-type antibiotic [42], apart from which little is known about bioactive compounds produced by the genus *Exiguobacterium*. The diversity of microbial metabolites may be partly underestimated, since each BGC does not necessarily produce only one metabolite, and growth conditions may change the metabolite produced by altering the expression of the same BGC [43]. Sequence identity at the level of genes in BGC producing similar compounds were reported to range between 58 and 80% [44]. By these criteria, the similarity to known BGC for our data is not very high, suggesting potential novelty.

The suggestion of metabolite novelty is encouraging since the RIT 452 extract contains compound(s) inhibiting a Gram positive clinical strain (MRSA) at lower concentrations (apparent MIC) than Gram negative clinical strains (Additional file 1: Table S1). Fractionation of the extracts using a non-clinical *S. aureus* strain shows that the activity is concentrated in the range of 95% acetonitrile, which hints at a relatively non-polar compound. Further “wet lab” experiments are needed to identify the bioactive compound(s) to verify the predictions made by bioinformatics analysis.

## Limitations

The active fraction contains more than one ionizable species and the chemical structure/s of the active compound/s are still unknown. MIC values and safety data of the isolated compound are also unknown.

## Supplementary Information

**Additional file 1: Figure S1.** A–D: Disc diffusion assay. RIT452 extracts (on discs) inhibit the growth of (A)* E. coli*, (B) *S. aureus*, (C) *P. aeruginosa*, and (D) *B. subtilis*. On each plate from top left to right the discs have 20 µl Tetracycline at 10 mg/ml (1), 10 µl extract (2), 20 µl extract (3), 40 µl extract (4), 60 µl extract (5), and 20 µl methanol as a negative control (6). (E) The inhibition zones in each case show a graded increase in the diameter of the zone of inhibition with increasing amounts of extract. Error bars from triplicate readings are shown and it can be seen that the linear range goes approximately up to 20 µL. **Table S1.** Estimated apparent MICs of the RIT 452 crude extracts computed according to the CLSI guidelines.

## Data Availability

The whole-genome project for *Exiguobacterium* sp. RIT452 is available in GenBank with the accession number QXJB00000000.

## Abbreviations

antiSMASH: Antibiotics and secondary metabolite analysis shell
ARTS: Antibiotic resistance target seeker
BGC: Biosynthetic gene cluster
CLSI: Clinical and Laboratory Standards Institute
ESI: Electrospray ionization
LB: Lysogeny broth
LC: Liquid chromatography
LCMS: Liquid chromatography-mass spectrometry
MIC: Minimum inhibitory concentration
MRSA: Methicillin resistant *Staphylococcus aureus*
MS: Mass spectrometry
R2A: Reasoner’s 2A
RIT: Rochester Institute of Technology
SEM: Scanning electron microscopy
TSA: Tryptic soy agar
TSB: Tryptic soy broth
ZOI: Zone of inhibition
