# Supplementary material for: Exiguobacterium sp. is endowed with antibiotic properties against Gram positive and negative bacteria
Source: BMC Res Notes. 2021 Jun 8;14:230. doi: 10.1186/s13104-021-05644-2 (PMC8186047; doi:10.1186/s13104-021-05644-2)
Supplement: Supplementary file 1 — Additional file 1: Figure S1. A–D: Disc diffusion assay. RIT452 extracts (on discs) inhibit the growth of (A) E. coli, (B) S. aureus, (C) P. aeruginosa, and (D) B. subtilis. On each plate from top left to right the discs have 20 µl Tetracycline at 10 mg/ml (1), 10 µl extract (2), 20 µl extract (3), 40 µl extract (4), 60 µl extract (5), and 20 µl methanol as a negative control (6). (E) The inhibition zones in each case show a graded increase in the diameter of the zone of inhibition with increasing amounts of extract. Error bars from triplicate readings are shown and it can be seen that the linear range goes approximately up to 20 µL. Table S1. Estimated apparent MICs of the RIT 452 crude extracts computed according to the CLSI guidelines. [file 13104_2021_5644_MOESM1_ESM.docx]

**
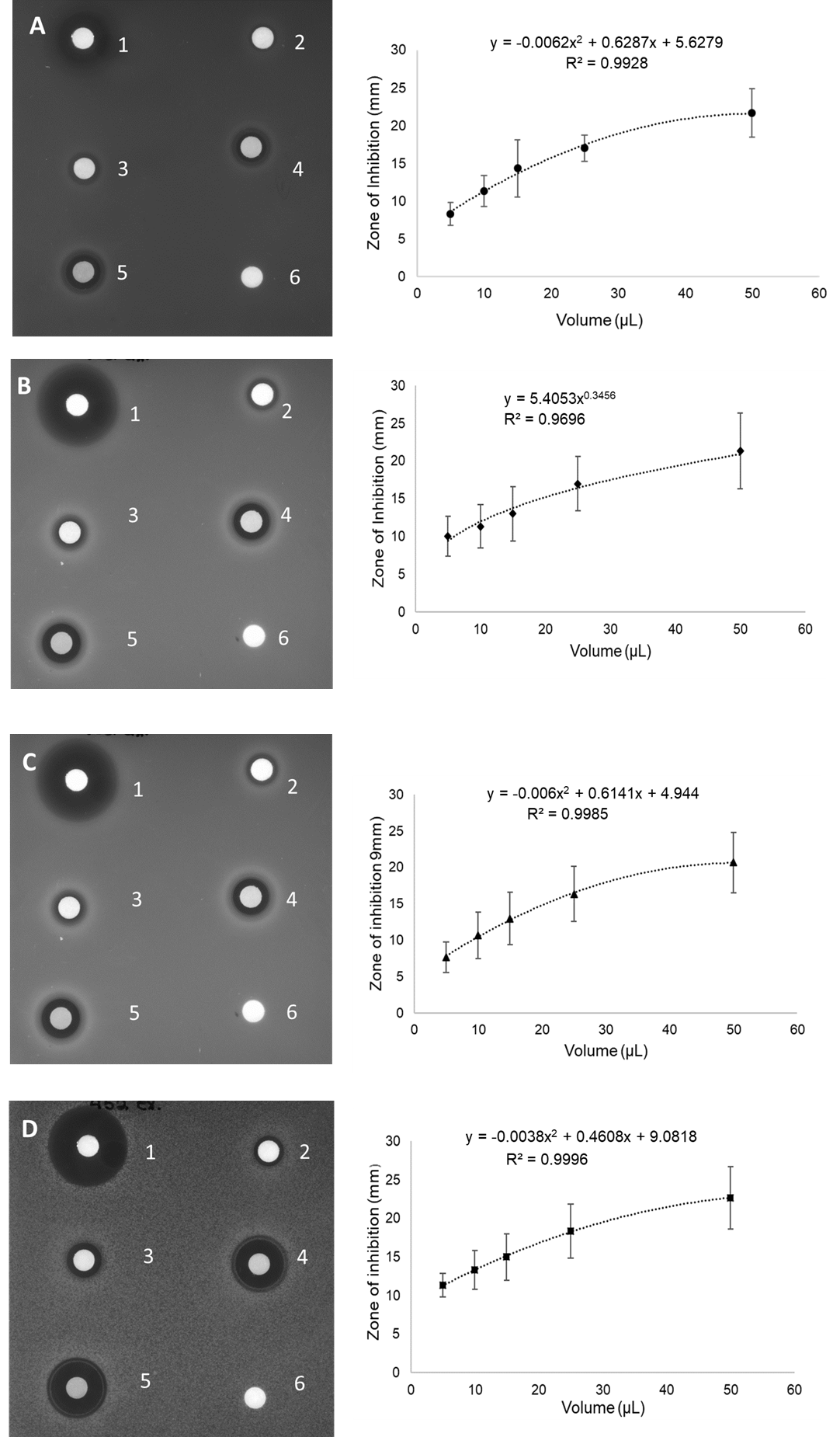
**

**Figures S1. A-D**: **Disc diffusion assay.** RIT452 extracts (on discs) inhibit the growth of (A) *E. coli*, (B) *S. aureus*, (C) *P. aeruginosa*, and (D) *B. subtilis*. On each plate from top left to right the discs have 20 µl Tetracycline at 10 mg/ml (1), 10 µl extract (2), 20 µl extract (3), 40 µl extract (4), 60 µl extract (5), and 20 µl methanol as a negative control (6). E) The inhibition zones in each case show a graded increase in the diameter of the zone of inhibition with increasing amounts of extract. Error bars from triplicate readings are shown and it can be seen that the linear range goes approximately up to 20 µL.

**Table S1.** Estimated apparent MICs of the RIT 452 crude extracts computed according to the CLSI guidelines. The multidrug resistant clinical isolates used were (relevant resistance genes listed after each isolate): MRSA USA300-FPR3757 (*mecA*), *E. coli* MCR1_NJ (*mcr-1, bla*_NDM-5_*, strA, strB, aac(6′)-Ib-cr, bla*_OXA-1_*, arr-3, sul1, sul2, tet*(A)), and *P. aeruginosa* AR-0230 (*aac(3)-Id*, *aadA2*, dfrB5, OXA-4, OXA-50, tet(G), VIM-2).

| Crude extract | MIC vs. MRSA | MIC vs. *E. coli* mcr1_NJ | MIC vs. P. aeruginosa AR-230 |
| --- | --- | --- | --- |
| RIT 452 | 3.125× | 6.25× | 6.25× |
